# Individual Rac GTPases Mediate Aspects of Prostate Cancer Cell and Bone Marrow Endothelial Cell Interactions

**DOI:** 10.1155/2011/541851

**Published:** 2011-06-27

**Authors:** Moumita Chatterjee, Linda Sequeira, Mashariki Jenkins-Kabaila, Cara W. Dubyk, Surabhi Pathak, Kenneth L. van Golen

**Affiliations:** ^1^The Laboratory for Cytoskeletal Physiology, Department of Biological Science, University of Delaware, Newark, DE 19716, USA; ^2^The Center for Translational Cancer Research, University of Delaware, Newark, DE 19716, USA; ^3^Biology Department, Lincoln University, Chester County, PA 19352, USA; ^4^The Delaware Biotechnology Institute, University of Delaware, 320 Wolf Hall, Newark, DE 19716, USA

## Abstract

The Rho GTPases organize the actin cytoskeleton and are involved in cancer metastasis. Previously, we demonstrated that RhoC GTPase was required for PC-3 prostate cancer cell invasion. Targeted down-regulation of RhoC led to sustained activation of Rac1 GTPase and morphological, molecular and phenotypic changes reminiscent of epithelial to mesenchymal transition. We also reported that Rac1 is required for PC-3 cell diapedesis across a bone marrow endothelial cell layer. In the current study, we queried whether Rac3 and RhoG GTPases also have a role in prostate tumor cell diapedesis. Using specific siRNAs we demonstrate roles for each protein in PC-3 and C4-2 cell adhesion and diapedesis. We have shown that the chemokine CCL2 induces tumor cell diapedesis via Rac1 activation. Here we find that RhoG partially contributes to CCL2-induced tumor cell diapedesis. We also find that Rac1 GTPase mediates tight binding of prostate cancer cells to bone marrow endothelial cells and promotes retraction of endothelial cells required for tumor cell diapedesis. Finally, Rac1 leads to *β*1 integrin activation, suggesting a mechanism that Rac1 can mediate tight binding with endothelial cells. Together, our data suggest that Rac1 GTPase is key mediator of prostate cancer cell-bone marrow endothelial cell interactions.

## 1. Introduction

Skeletal metastases represent a major clinical problem for men suffering from prostate cancer (PCa). Nearly 80% of men who die from this disease have significant spread of the cancer to bone [1, 2]. Like all cancers, PCa cells must successfully complete a series of ordered steps, known as the metastatic cascade, to form a distant tumor [3, 4]. One key step in the PCa metastatic cascade is the process of extravasation from the circulation into the bone microenvironment [5, 6]. The process of PCa cell extravasation can be subdivided into a number of substeps, which include arrest, binding, adhesion, and spreading on bone marrow endothelial cells, migration along the endothelial barrier, tumor cell diapedesis, and invasion into the bone stromal compartment [6–8]. Although many of these substeps have been well studied for leukocyte extravasation, relatively little is known about the process of PCa tumor cell extravasation across a bone marrow endothelium (reviewed in [9]). 

The Rho GTPases are a group of proteins that comprise a subfamily of the Ras-superfamily of monomeric GTP-binding proteins that act as molecular switches regulating the cytoskeleton promoting cell migration [10–14]. Furthermore, the Rho proteins are implicated in cancer progression and metastasis (reviewed in [15]). Previously, we have suggested potential roles for individual Rho GTPases in PCa extravasation [16–18]. Specifically, we demonstrated that RhoC GTPase is required for invasion in response to insulin-like growth factor I and type I collagen [16, 18]. Upregulation of the integrin heterodimer *α*2*β*1 in LNCaP cells selected for their ability to bind to type I collagen led to increased RhoC activation and cellular invasion upon integrin ligation [19, 20]. Downregulation of RhoC in PC-3 human PCa cells through introduction of either a dominant negative (dn)RhoC or a RhoC-specific shRNA led to a significant decrease in the cells ability to invade either collagen or Matrigel-coated filters [16, 18]. However, these cells underwent changes reminiscent of epithelial to mesenchymal transition (EMT). Concordant with EMT, the cells displayed increased random linear motility, which was due to increased and sustained levels of Rac1 GTPase expression and activation. Further, we demonstrated that active Rac1 GTPase is required for PC-3 cell diapedesis across a BMEC layer [18].

The Rac GTPase branch of the Rho subfamily is comprised of four members, Rac1, Rac2, Rac3, and RhoG. Rac1 and RhoG are ubiquitously expressed, while Rac2 is primarily expressed in hematopoietic cells, and Rac3 is expressed mainly in nervous tissue but can be found expressed at lower levels in most other tissues (reviewed in [21]). Seminal experiments demonstrated a role for Rac1 in the formation of lamellipodia [11, 13, 14, 22, 23]. Recent evidence suggests a role for Rac3 GTPase in cellular adhesion and neurite outgrowth [24–26]. RhoG GTPase has been shown to signal in a parallel pathway to Rac1, being regulated by some of the same upstream regulatory proteins such as Vav2 and activating some of the same downstream effectors as Rac1 [27]. RhoG has also been shown to act as a hierarchical GTPase; activation of RhoG can lead to the activation of Rac1 through direct interaction with the Dock180-ELMO [28]. Therefore, Rac1-mediated cell migration can be regulated through direct activation of RacGEFs or via RhoG GTPase [29].

 Rac1 GTPase plays an intimate role in monocyte and macrophage diapedesis [30]. Monocytes are recruited to the sites of inflammation via stimulation by the chemokine CCL2 (a.k.a. MCP-1) [31]. Binding of CCL2 to its putative receptor CCR2 leads to clustering of the novel actin regulatory protein PCNT1 to the cells leading edge and regulation of migration through activation of Rac1 [30]. Activation of Rac1 is required to form lamellipodia, which in turn is required for monocytes to sense junctions between endothelial cells [30]. BMECs from PCa patients secrete high levels of CCL2 [32]. Stimulation of PC-3 cells with CCL2 results in activation of Rac1 GTPase and EMT consistent with what is observed when RhoC activity is downregulated through introduction of a dnRhoC or shRNA to RhoC [16–18]. Furthermore, CCL2 stimulation drives PC-3 tumor cell diapedesis across a BMEC layer via PCNT1 [17]. 

Levels of Rac1 and Rac3, but not Rac2, are shown to be increased in prostate cancer patient samples compared to normal prostate [33]. However, the role that these GTPases play in PCa tumor cell metastasis has not been thoroughly studied. Similarly, there is no information on the contribution of RhoG to PCa progression. In the current study we investigate the roles of Rac1, Rac3, and RhoG GTPases in the process of prostate tumor cell diapedesis across a bone marrow endothelial cell layer. All three Rac proteins have an influence on tumor cell diapedesis across a bone marrow endothelial cell monolayer. Further, we demonstrate that Rac1 GTPase has a significant effect on PCa cell diapedesis, while Rac3 has a negative effect on tumor cell diapedesis. In addition, RhoG has a partial effect on CCL2-stimulated diapedesis. Finally, Rac1 regulates binding of prostate cancer cells to the bone marrow endothelial cells. Our data suggest that Rac1 is required for the activation of *β*1 integrins leading to binding of the prostate cancer cell to the BMEC. This is the first study to demonstrate roles for different isoforms of Rac GTPase in the PCa metastatic phenotype.

## 2. Materials and Methods

### 2.1. Cell Lines and Cell Culture

PC-3 PCa cell lines were obtained from American Type Culture Collection (Manassas, Va) and maintained in Ham's F-12 medium with 1.5 g/L sodium pyruvate, 2 mM L-glutamine, and 10% FBS (Invitrogen/Gibco, Carlsbad, Calif). C4-2 cells were a gift from Dr. Robert Sikes (University of Delaware) and maintained in T-medium containing 10% FBS (Invitrogen/Gibco). Human bone marrow endothelial cells (BMECs) were a gift from Dr. Graca Almeida-Porada (University of Nevada School of Medicine, Reno, Nevada). Cultures were maintained in Medium 199 with Earles's salts, L-glutamine, 2,200 mg/L sodium bicarbonate, 25 mM HEPES (Invitrogen/Gibco) buffer, 10% FBS, 1% pen/strep, endothelial cell growth supplement (BD Biosciences, Bedford, Mass), and 7500 u/500 mL media of heparin (Sigma-Aldrich, St. Louis, Mo). All cell lines were maintained at 37°C in a 90% : 10% air : CO_2_ incubator. C3 exotransferase was introduced into cells as previously described using a lipid transfer-mediated method [34] and treated for 2 h before analysis. Rac1 inhibitor NSC23766 (Calbiochem, San Diego, Calif) treatment was performed by adding directly to tissue culture medium to a final concentration of 100 *μ*M 1 h prior to analysis. Prostate cancer cells were stimulated with 100 ng/mL recombinant human (rh)CCL2 (MCP-1) in tissue culture medium (Millipore-Chemicon Inc., Billeceria, Mass) for 30 min during the Rac activation assays and kept in the presence of the chemokines during the diapedesis assays.

### 2.2. siRNAs

Specific siRNAs for human Rac1 and Rac3 GTPases were a gift from Dr. Marc Symons and described previously [26]. RhoG siRNA and scrambled control siRNAs were synthesized by integrated DNA technologies. RhoG siRNA target sequences were (1) 5′-TGCCCTGATGTGCCCATCCTGCTGGTGGG-3′ and (2) 5′-ACGTGCCTGCTCATCTGCTACACAACTAA-3′. The Rac1, Rac3, and RhoG siRNA duplexes were formed by adding 30 *μ*L of each RNA oligo solution together with 15 *μ*L of 5x annealing buffer (100 mM NaCl and 50 mM Tris-HCl pH 7.5) to give a final volume of 75 *μ*L and a final concentration of 20 *μ*M; incubated for 2 min in water bath at 95°C; allowed to cool to room temperature. Additional experiments were performed using ON-TARGET plus SMARTpool siRNAs Rac1, Rac3, and RhoG siRNAs that were obtained from Dharmacon (Dharmacon/Thermo Scientific, Layfette, Colo). siRNAs were transfected into prostate cancer cells using FuGene6 (Roche, Indianapolis, Ind) or GeneSilencer Reagent (Genlantis, San Diego, Calif) per the manufacturers instructions and cells used 72 h after transfection. For rescue experiments, mutations were generated using the QuickChange II Site-Directed Mutagenesis kit (Stratagene) according to the manufactures recommendations. To fully abolish the effect of siRNAs, two nucleotides in the siRNA-targeted area were changed in both Rac3 and RhoG GTPases. To create a RhoG fast cycling mutant, glutamine 63 was converted to lysine.

### 2.3. Reverse Transcriptase and Real-Time Quantitative PCR

Total RNA was harvested from cells and converted to cDNA as previously described [16]. PCR primers were designed using the primer design feature on the Evocycler PCR program (Evogen Ltd., UK). Primer design parameters were set to optimally produce PCR products between 100 and 150 bp in size. Primer sequences are found in Supplemental Table 1 (see Table  1 in Supplementary Material available online at doi:10.1155/2011/541851). RT-PCR was performed on an Evocycler EPx (Evogen Ltd.) using Fast SYBR Green chemistry (Applied Biosystems Inc., Foster City, Calif) per the manufacturers recommendations for 30 cycles (98°C for 15 s, 67°C for 15 s, and 72°C for 30 s), and PCR products visualized on a virtual gel and band intensities were normalized to GAPDH using the Evocycler PCR program. 

For quantitative (q)PCR, RNA was isolated from the cell lines using TRIzol Reagent (Invitrogen, Carlsbad, Calif). cDNA was synthesized from this RNA using the Promega Reverse Transcription kit (Promega Corp., Madison, Wis). Appropriate primers (Integrated DNA Technologies, Inc., Coralville, Iowa) were diluted to a final concentration of 10 *μ*M. The cDNA synthesized from the isolated RNA was diluted to a final concentration of 4 ng/*μ*L. Reactions were prepared as a bulk “master mix” using the ABI SYBR Green PCR Master Mix (Applied Biosystems Inc., Foster City, Calif) for each target gene/primer pair used. Three no-template controls were included for each primer pair being used. A 5 *μ*L aliquot of cDNA was pipetted into each well of the ABI 96-well plate, and 20 *μ*L of the reaction master mix was added to it. Plates were covered with ABI adhesive cover, centrifuged at 1000 rpm to mix the contents, and run on an ABI 7000 real-time qPCR machine housed in the Center for Translational Cancer Research (University of Delaware).

### 2.4. Tumor Cell Diapedesis Assays

Tumor cell diapedesis assays were performed as previously described [18]. Briefly, 100,000 HBME cells were added to the top chamber of either uncoated or Matrigel-coated Transwells 24 h prior to the assay and allowed to form a confluent monolayer. PC-3 and C4-2 cells were harvested, labeled with Calcein AM (Invitrogen/Molecular Probes) per manufacturers recommendations, and resuspended in serum-free medium containing 0.1% BSA at a concentration of 3.75 × 10^5^ cells/mL, and 0.5 mL was added to the top chambers. The chambers were incubated for 24 h at 37°C in a 10% CO_2_ incubator. Medium was aspirated from the top chamber, and excess Matrigel and cells were removed from the filter using a cotton swab. Filters were cut away from the inserts, mounted on microscope slides, and visualized on a fluorescent microscope and number of invaded cells counted.

### 2.5. Rac GTPase Activation Assay

Activation of total Rac GTPase proteins was performed using a GLISA pan-Rac activation assay kit (Cytoskeleton Inc., Denver, Colo) as previously described [18]. Briefly, prostate cancer cells were grown to 75% confluence in a 100 mm dishes and serum starved for 24 h. On the day of the assay, cells were harvested using nonenzymatic cell dissociation buffer (Sigma-Aldrich), washed twice with ice-cold PBS, and resuspended in 65 *μ*L GLISA lysis buffer. Protein lysates were transferred to ice-cold 1.5 mL centrifuge tubes and clarified by centrifugation at 10,000 rpm for 2 min. Protein concentrations were determined using the supplied Precision Red advance protein assay and 1.0 mg/mL protein used for the GTPase activation assay per manufacturers recommendations. After antibody and horseradish peroxidase detection reagent incubation, signals were detected on a Benchmark Plus microplate spectrophotometer at 490 nm (Bio-Rad Laboratories, Hercules, Calif).

### 2.6. Atomic Force Microscopy

All AFM experiments were conducted with a Bioscope II (Vecco, Santa Barbara, Calif) using silicon-nitride tips (Vecco; spring constant 0.06 N/m). Unbinding force measurements were conducted with tips functionalized with collagen or fibronectin (Becton-Dickinson, Franklin Lakes, NJ) at concentrations of 50 *μ*g/mL and 15 *μ*g/mL, respectively. Likewise, 35 mm tissue culture dishes (Corning Inc., Corning, NY) were coated with collagen or fibronectin and sterilized under ultraviolet light overnight. PC-3 cells were transfected with siRNA specific for Rac1, Rac3, or RhoG using FuGene6 (Roche) or GeneSilencer Reagent (Genlantis) and plated on the prepared dishes 8 h prior to experimentation. BMECs were cultured in RPMI 1640 media (Hyclone/Thermo Scientific) supplemented with 10% FBS. The functionalized AFM tip was dropped onto a single live BMEC cell and after attachment was verified, the loaded tip was gently lowered onto the center of a PC-3 cell. The unbinding force interaction between the two live cells was measured. The unbinding force is the force required to separate two adhesion molecules and is measured in picoNewtons (pN). The number of events for a particular unbinding force is the number of molecules separated at each force. Specifically, 250 unbinding events were captured per cell site with 4 areas probed per cell, and 3 separate cells were probed per treatment. Force curves were generated at a frequency of 1 Hz in a relative trigger mode. 

AFM stiffness measurements were based on recording the elastic response of cells, BMECs and PC-3s using an AFM tip. The AFM was operated in the force-volume mode for recording a set of loading/unloading load displacement curves at a frequency of 1.03 Hz and a forward/reverse velocity of 4.11 *μ*m/sec. The resultant measurement is the dynamic elastic modulus (a.k.a. the Young's modulus), which measures the stiffness of the cell. The Young's modulus is the ratio of stress to strain and is thus represented by units of pressure, Pascals (Pa). Cell stiffness changes are due to morphologic changes resulting from alterations in cytoskeletal structure (reviewed in [35]). The elastic modulus was measured with individual BMECs, individual PC-3 cells, and the duo: PC-3 cells attached to plated BMECs and BMECs attached to plated PC-3 cells. The elastic modulus for the BMEC/PC-3 combinations was generated for the plated cell, and the attached cells separately. Each force-volume map consists of 256 data points per sample site with 3 separate sites measured per experimental condition, 3 separate times.

### 2.7. Transendothelial Electrical Resistance (TEER)

Transendothelial electrical resistance (TEER) measurements were done using Epithelial Voltohmmeter (EVOM; World Precision Instruments Inc., Sarasota, Fla) following manufacturers directions. Briefly, BMECs were plated at a concentration of 1.3 × 10^6^ cells/mL on 12-well 0.4 *μ* polycarbonate membrane inserts (CLS3401; Corning Transwell) and were maintained until day 4 (we determined empirically that the TEER for the BMEC monolayer was optimum on day 4 after plating due to maturation of cell junctions). On day 4, tissue culture medium was removed from the top chamber, an equal concentration of PC-3 cells was added to the BMEC monolayer and TEER measured at specified intervals.

### 2.8. Fluorescence-Activated Cell Sorting (FACS) Analysis

Prostate cancer cells were cultured in T25 flasks (Corning Inc., Edison, NJ), detached, washed, and resuspended in 5% bovine serum albumin (BSA; Sigma-Aldrich) in phosphate buffered saline (PBS; Sigma-Aldrich). All washes and resuspensions were also performed in 5% BSA containing PBS. One set of control and siRac1-transfected prostate cancer cells were each further treated with CCL2 (100 ng/mL) for 30 min, washed, and resuspended. The several states of *β*1 activation were queried with two conformation-sensitive antibodies N29 (BD Biosciences, Franklin Lakes, NJ) and HUTS-21 (BD Biosciences) in addition toa total *β*1 conformation-insensitive antibody, MAR4 (Chemicon, Billerica, Mass). All antibodies were used at a final concentration of 10 *μ*g/mL, and all incubations were conducted in the dark and at 37°C. Cells were analyzed using an FACS Calibur cytometer (BD Biosciences), equipped with 488 nm and 633 nm lasers. Analyses were performed on 10,000-gated events, and the numeric data were processed with Cellquest software (Becton Dickinson).

### 2.9. Statistical Analysis

All experiments were performed a minimum of three separate times with individual transfections consisting of no less than three replicates per experiment. Statistical analysis of the combine experiments was performed using GraphPad Prism and by the University of Delaware College of Agriculture and Natural Resources Statistics Laboratory. A one-way ANOVA analysis was used with Bonferroni's post hoc analysis for comparison between multiple groups. A Students *t*-test was used for comparison between two groups. Significance was defined as a *P*  value < .001. Data is represented as mean ± standard deviation.

## 3. Results

### 3.1. Active Rac GTPases Affect Prostate Cancer Cell Transendothelial Migration

**Figure 1:**
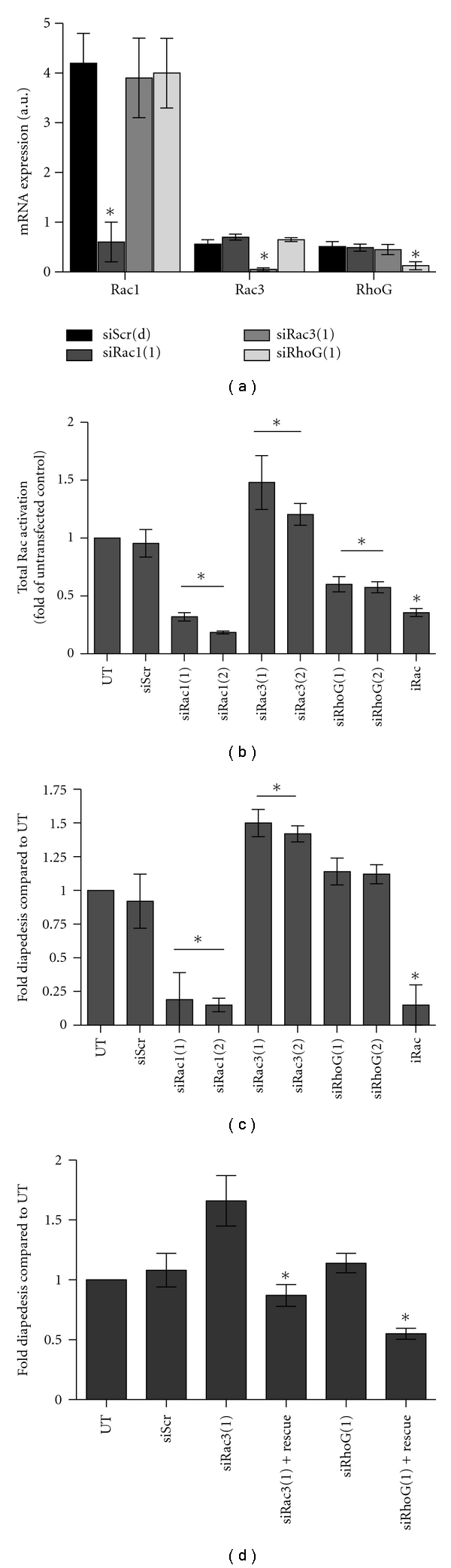
Effect of Rac depletion on total Rac activation and tumor cell diapedesis across a bone marrow endothelial cell monolayer. (a) Rac isoform expression and isoform-specific depletion in PC-3 cells. Individual siRNAs (1) and (2), specific for Rac1, Rac3, or RhoG, were compared. Messenger RNA was harvested and SYBR green-based qPCR performed using primers specified in Supplemental Table 1. Relative expression levels were normalized to GAPDH expression from the corresponding sample and expressed as arbitrary units (a.u.). (b) is the effect of the RacGEF inhibitor NSC23766 (iRac), siRNA specific for Rac1, Rac3, and RhoG, or scrambled control (siScr) on total Rac activation. Cells were treated with 100 *μ*M NSC23766 for 1 h or 20 *μ*M siRac1, siRac3, or siRhoG. Activation of total Rac was performed using GLISA. Cells treated with iRac or transfected with siRNA to Rac isoforms were compared with untransfected (UT) and representative siRNA-scrambled control (siScr). Each analysis was performed in triplicate with individual transfections. (c) BMECs were layered onto a Matrigel-coated filter and allowed to form a monolayer; 0.5 mL of a suspension of 3.75 × 10^5^  PC-3 cells/mL were added to the BMECs and allowed to undergo diapedesis for 24 h. Treated and transfected cells were compared with untransfected or scrambled controls. (d) Introduction of an RNAi-insensitive Rac3 into siRac3-treated PC-3 cell. Shown in all four panels is the mean ± S.D. of at least triplicate analysis with significance being *P < .001. Noncapped lines above the bars represent that the siRNA group is significantly different from controls.

Previously, we demonstrated that Rac1 GTPase was required for tumor cell transendothelial cell migration. However, we did not thoroughly explore if other Rac family members contributed to diapedesis [16–18]. The expression of Rac1 and Rac3 GTPases is increased in PCa patient tumors; however, it is unknown if RhoG is expressed [33]. Using quantitative (q)PCR, we demonstrate detectable message for Rac1, Rac3 and RhoG in the PC-3 cells. As shown in Figure 1(a), normalized Rac1 mRNA expression levels were on average 8-fold higher than both Rac3, and RhoG, suggesting that Rac1 is the predominant Rac GTPase expressed in the PC-3 cells. Expression levels were confirmed when the products of a semiquantitative PCR were visualized by virtual gel (Supplemental Figure 1). Band intensity for each product on the virtual gel is automatically normalized to the corresponding GAPDH. Similar expression levels were observed for C4-2 prostate cancer cells (Supplemental Figure 2(a)). Due to the lack of specific antibodies, particularly for Rac3, protein expression levels were not assessed by Western blot analysis. 

To elucidate the role of each Rac protein in transendothelial cell migration, we selectively downregulated the expression of Rac GTPase isoforms using siRNA. Figure 1(a) is the results of isoform-specific Rac message depletion using siRNA duplexes. Expression of each Rac mRNA was significantly reduced by a minimum of 80% compared to PC-3 cells treated with an appropriate scrambled control. Each siRNA specifically reduced its target without affecting other Rac GTPases or affecting cell growth (growth data not shown). Similar results were seen when alternate siRNAs were used for each Rac isoform. 

PC-3 cells were treated with the pharmacologic RacGEF inhibitor NSC23766 (iRac) or Rac-specific siRNA and the effect on total Rac activity determined (Figure 1(b)). As expected, both the NSC23766 inhibitor and Rac1-specific siRNA reduced total active Rac levels by ~60% compared to untransfected control. Interestingly, knockdown of RhoG led to a significant 45% reduction in total Rac activity suggesting that RhoG may activate Rac1 during physiologic process such as diapedesis. In contrast to Rac1 and RhoG, knockdown of Rac3 resulted in a significant 52% increase in total Rac activity compared to control. 

Since Rac GTPases are required for transendothelial cell migration across a BMEC layer [18], we next tested the individual role of Rac1, Rac3, and RhoG in PCa diapedesis across a BMEC layer. As expected, downregulation of Rac1 led to a significant decrease in diapedesis (Figure 1(c)). However, inhibition of RhoG and Rac3 had no effect on inhibiting tumor cell diapedesis. In contrast to Rac1, depletion of Rac3 led to a 70% increase in transendothelial migration, suggesting that Rac3 limits PCa diapedesis similar to what has been shown for RhoA in PCa invasion [18]. The increase in diapedesis observed when RhoG was depleted approached but did not achieve significance compared to untransfected or scrambled controls. Figure 1(d) demonstrates that expression of a siRNA-resistant Rac3 in Rac3-downregulated cells results in a significant decrease in diapedesis. Similarly, re-expression of RhoG led to a significant decrease in diapedesis compared to cells depleted of RhoG. This suggests a negative effect of Rac3 and possibly RhoG on tumor cell diapedesis. Supplemental Figure 2(b) demonstrates a similar trend for the C4-2 prostate cancer cells. Depletion of Rac1 led to a significant decrease in transendothelial cell migration. However, depletion of Rac3 or RhoG increased tumor cell diapedesis. Rescue experiments reversed the trends of the siRNAs in the C4-2 cells.

### 3.2. The Chemokine CCL2 Stimulates Diapedesis via RhoG GTPase

**Figure 2:**
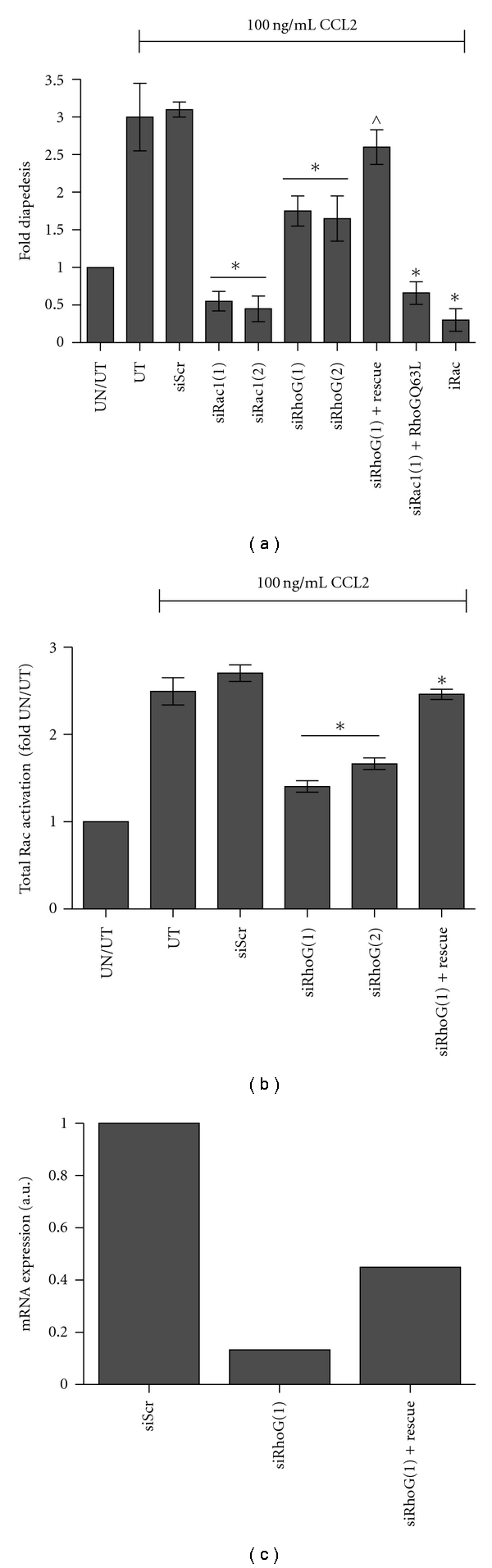
Effect of Rac depletion on tumor cell diapedesis across a BMEC monolayer. (a) PC-3 cells were treated with 100 ng/mL CCL2 in a diapedesis assay. Control untransfected (UT) and siRNA control (siScr) cells demonstrated increased diapedesis compared with untreated/untransfected (UN/UT) PC-3 cells. The ability of cells to undergo CCL2-stimulated diapedesis after depletion of Rac1, RhoG, or treatment with iRac was compared to UT and siScr. Rescue experiments of RhoG-depleted cells were performed by the introduction of a siRNA-insensitive RhoG. Rac1-depleted cells were rescued with the introduction of fast cycling RhoG (RhoGQ63L). (b) Depletion of RhoG led to a decrease of total Rac activation in PC-3 cells treated with 100 ng/mL CCL2. Rescue experiments were performed by introducing a siRNA-insensitive RhoG GTPase. Shown are means ± S.D. of at least triplicate analysis representing individual transfections, with significance being *P* < .001; (*) signifies a significant difference between siRNA-transfected cells and stimulated controls, while (^*∧*^) signifies a significant difference between siRNA-transfected and -rescued cells.

The chemokine CCL2 is produced by BMECs and stimulates Rac1-mediated tumor cell diapedesis [17, 32]. Since RhoG appears to have an effect on Rac activation, we next set out to determine if CCL2-stimulated diapedesis could be affected by depletion of RhoG. As shown in Figure 2(a), CCL2 treatment increased diapedesis 3-fold across a BMEC layer in untransfected (UT) and siScr control cells compared to untreated/untransfected cells (UN/UT). Contrary to what we observed for unstimulated diapedesis in Figure 1(c), there was an approximate 45% decrease in PC-3 diapedesis across the endothelial cell layer when RhoG was depleted using siRNAs (*P* < .001). Similarly, direct depletion of Rac1 or treatment with the inhibitor NSC23766 led to a significant decrease in transendothelial cell migration. Introduction of a siRNA-resistant RhoG fully rescued CCL2-induced diapedesis in RhoG-depleted cells. However, introduction of a RhoGQ63L fast cycling mutant did not rescue the cells ability to cross an endothelial cell layer when Rac1 was depleted. Finally, Supplemental Figure 2(c) demonstrates that CCL-2-induced diapedesis is inhibited in C4-2 cells when RhoG is depleted. Again, introduction of a siRNA-resistant RhoG fully restores the cells ability to cross the BMEC layer. 

Figure 2(b) demonstrates that CCL2-induced total Rac activation is decreased by ~40% when RhoG is depleted from the PC-3 cells, suggesting that CCL2 may activate Rac1 directly and also indirectly through RhoG GTPase. Concordant to what is observed in the diapedesis assay, introduction of a siRNA-resistant RhoG restores actives levels of total Rac similar to controls. Restoration of Rac activity and PCa diapedesis in the rescue experiments were not due to overexpression of nonphysiologic levels of ectopic RhoG. As shown in Figure 2(c), during rescue, mRNA levels of RhoG were increased 4-fold over the RhoG-depleted cells. These expression levels were still well under what is observed for the siScr control cells. Similar results were observed for the C4-2 cells and in the RNAi-insensitive Rac1 rescued cells. On average, an ~70% transfection efficiency was observed for each construct in both the PC-3 and C4-2 cells.

### 3.3. Rac1 GTPase Mediates the Interaction between PC-3 Cells and BMECs

**Figure 3:**
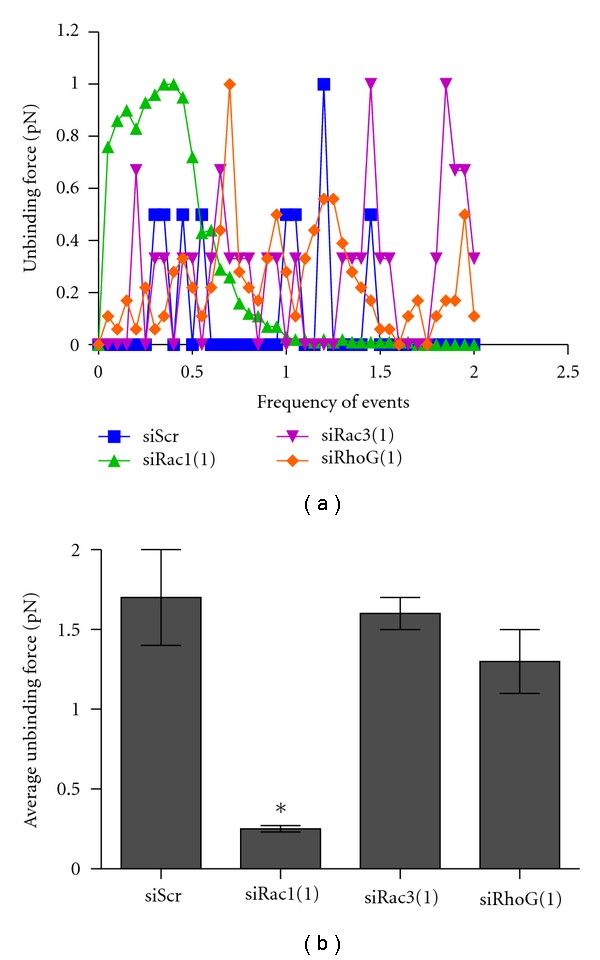
Interaction of prostate cancer cells with bone marrow endothelial cells BMECs was attached to the AFM tip and the unbinding forces of PCa cells measured. PC-3 cells were transfected with siRNAs specific for individual Rac isoforms. Shown are the results from one set of siRNAs. (a) Effect on the frequency of unbinding events and forces (pN) occurring between BMECs and PC-3 cells after depletion of each Rac isoform. (b) Average unbinding force occurring between BMECs and PC-3 cells. The average unbinding force is the physical force required to pull two adhered cells apart. Data are compiled from 3000 data points and are the mean ± S.D. with significance being *P < .001.

We previously demonstrated that downregulation of Rac1 does not significantly affect PC-3 cell binding to BMECs [18]. However, anecdotal evidence suggested that Rac1 depletion leads to decreased binding strength of the PC-3 cells to BMECs. To quantitate binding strength, we used atomic force microscopy (AFM) to measure the unbinding force of PC-3 cells bound to BMECs after Rac1, Rac3, or RhoG depletion. For the siScr control, siRac3- and siRhoG-treated PC-3 cells, a number of individual unbinding events occurred over time (Figure 3(a)) suggesting tight binding of multiple adhesion molecules is involved in cell-cell contact. In contrast, down-regulation of Rac1 led to a significant decrease in the number and frequency of unbinding events that occurred, suggesting fewer and weaker cell-cell contacts. Figure 3(b) shows that depletion of Rac1 led to a significant average 85% decrease in the unbinding force of the PCa cells to the bone marrow endothelial cells. Interestingly, downregulation of RhoG did not affect the ability of the PC-3 cells to bind to the BMECs, suggesting that RhoG activation of Rac1 is not involved in cell-cell binding.

**Figure 4:**
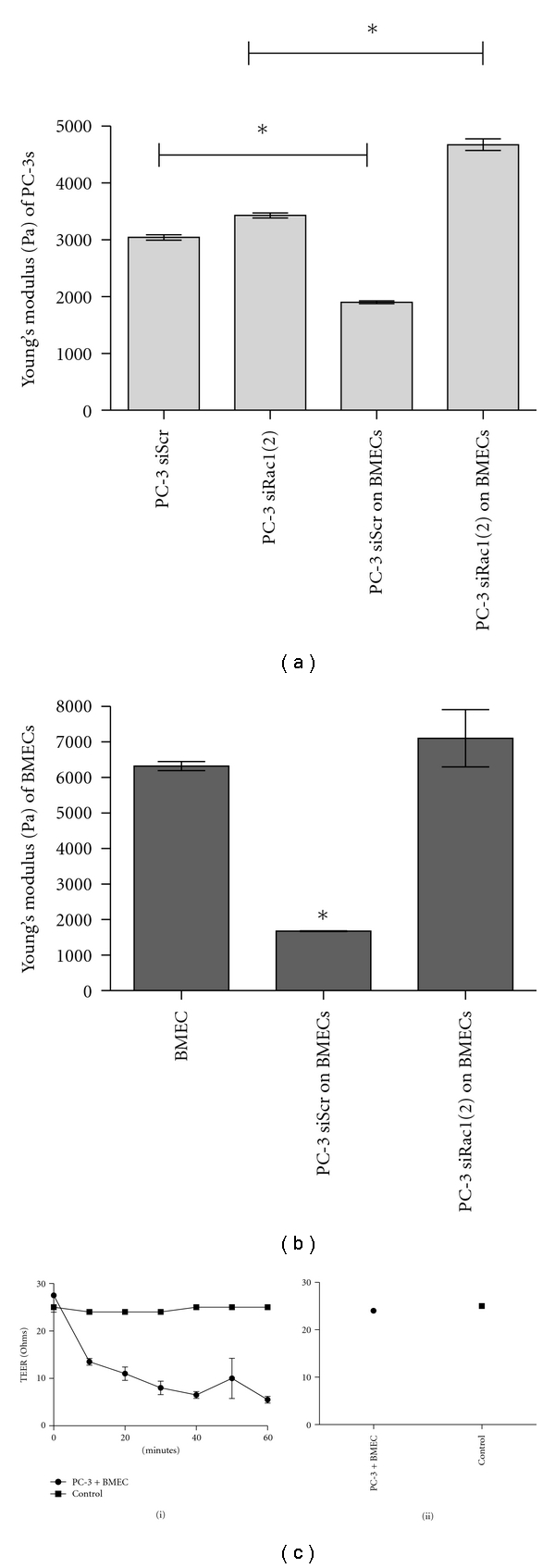
Bone marrow endothelial cells react to PCa cell binding. (a, b) are measurements of the dynamic elastic modulus or elasticity of PC-3 and BMECs in contact with one another. Elasticity is given as the Young's modulus and is a ratio of cell stress and strain and is measured in Pascals. BMECs were grown as a monolayer and control (siScr) or siRac1 expressing PC-3 cells were allowed to bind to the BMEC monolayer, and the elasticity of the PC-3 cells (a) and the BMECs (b) was measured by AFM. Data are the result of over 10,000 data points and represented as mean ± S.D. with significance being *P < .001. (c) are measurements of transendothelial electrical resistance (TEER). BMECs were grown on a monolayer, PC-3 cells were added to the monolayer, and the electrical resistance was measured every 10 min up to 1 h (i) and the final measurement at 24 h (ii).

In a system resembling initial contact during diapedesis, PC-3 cells were allowed to bind to a BMEC monolayer, and the dynamic elastic modulus (a.k.a. Young's modulus) was measured using AFM. Because of the pronounced effect of Rac1 depletion on PCa cell adhesion to BMECs seen in Figure 3, we compared siScr control and siRac1-transfected PC-3 cells. Figure 4(a) shows that the elasticity was essentially unchanged for the siRNA-scrambled control and siRac1 PC-3 cells alone. Compared to the unbound cells, the siRNA control PC-3 cells became more elastic (a decrease in the Young's modulus) when bound to BMECs suggesting that they begin to spread onto the endothelial cell monolayer. In contrast, the PC-3 cells transfected with siRNA to Rac1 were significantly less elastic (increase in the Young's modulus) than the unbound PC-3 cells when bound to BMECs suggesting that they remain in a rounded configuration as we previously reported [16].  Figure 4(b) compares the elasticity of the cells in the BMEC monolayer when engaged by the PC-3 cells. BMECs had a significant 30% increase in elasticity when in contact with control PC-3 cells suggesting reorganization of the actin cytoskeleton. In contrast, the BMECs had no change in theirdynamic elastic modulus when bound to Rac1-depleted PC-3 cells. 

In a variation of this experiment, we allowed individual BMECs to come into contact with a PC-3 cell monolayer (Supplemental Figure 3). Again, the elasticity of the PC-3 cells was essentially unchanged due to downregulation of Rac1. There was a significant and consistent 30% increase in elasticity of the BMECs when they came in contact with the control PC-3 cells. However, there was no change in elasticity of the BMECs when they came into contact with PC-3 cells that had depleted Rac1. 

Figure 4(c)(i) demonstrates a marked change in transendothelial electrical resistance (TEER) across the BMEC layer. TEER is a measure of the integrity of tight junctions between cells. Decreased TEER is indicative of cellular retraction. Addition of PC-3 cells to a confluent BMEC layer led to a significant, time-dependent decrease in TEER. TEER levels were fully restored, in a time-dependent manner by 24 h (Figure 4(c)(ii)). Taken together these data suggest that the BMECs undergo cytoskeletal changes that influence their elasticity when they interact with PC-3 cells.

### 3.4. Active Rac1 GTPase Leads to Stimulation of *β*1 Integrins

**Figure 5:**
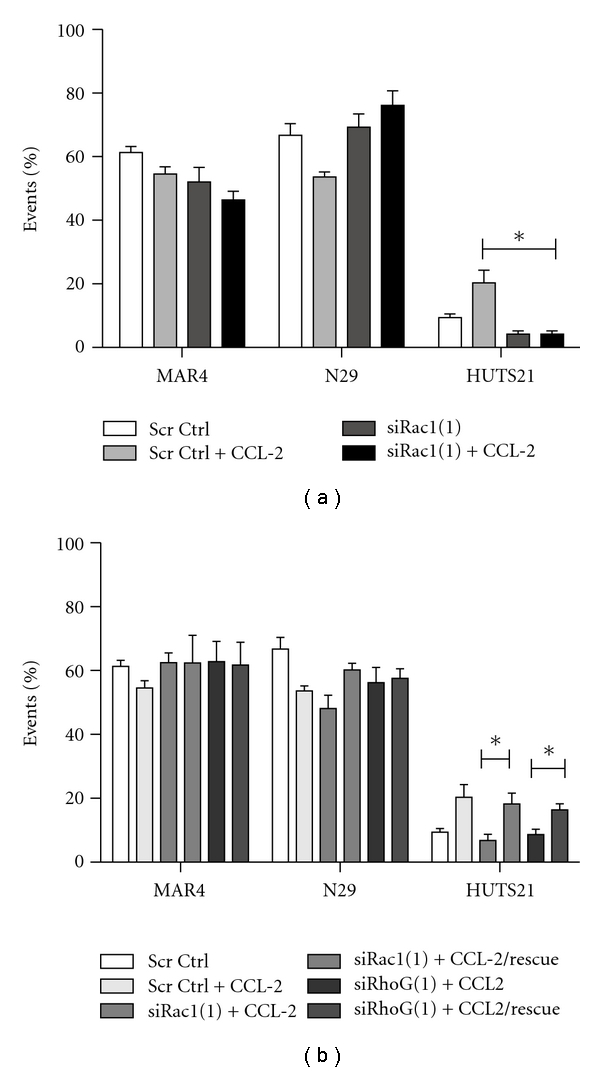
Effect of Rac1 GTPase on *β*1 integrin activation. (a) Comparison of total, partially and fully activated *β*1 integrinsin control and Rac1 depleted PC-3 cells as assessed by FACS analysis. Cells were transfected with either siRNA-scrambled control or Rac1 siRNA(2) and left unstimulated or treated with 100 ng/mL CCL2. FACS analysis was performed after incubating fixed cells with the antibodies MAR4 (for total *β*1 integrin), N29 (for partially active *β*1 integrin), and HUTS21 (for fully active *β*1 integrin). (b) is a rescue experiment demonstrating that introduction of either an RNAi-insensitive Rac1 or RhoG GTPase leads to restoration of active *β*1 integrin levels in CCL2-treated PC-3 cells. Shown are the results of triplicate experiments showing the percentages of 10,000 gated events with significance being *P < .001. Capped lines signify a comparison and significance between siRNA-depleted cells and cells rescued with siRNA-insensitive constructs.

Active Rho GTPases are known to lead to expression and activation of integrins [36]. Two integrin heterodimers are associated with binding to VCAM-1 and ICAM-1 on endothelial cells, *α*4*β*1 and *α*L*β*2, respectively. With this in mind, we set out to determine if Rac1 GTPase influenced the activation state of integrins leading to BMEC binding. Since *β*2 integrins are not associated with prostate cancer and the role of the *β*1 integrins is established in PCa/BMEC interactions [6, 8, 9], we focused on the expression and activation of the *β*1 subunits. To determine this fluorescence-activated cell sorting (FACS), analysis was performed using a set of antibodies that recognize total and active levels of the *β*1 subunit. The MAR4 antibody recognizes total *β*1 integrin subunit regardless of activation state. The *α*4*β*1 heterodimer can exist in 3 conformations, closed headpiece/bent (inactive), closed headpiece/extended (partially activated, recognized by the N29 antibody), and open headpiece/extended (fully active, recognized by the HUTS21 antibody). Figure 5(a) demonstrates that unstimulated PC-3 cells have similar levels of total and partially activated *β*1 integrin as compared to Rac1-depleted PC-3 cells. When the cells were treated with CCL2, thus leading to increased Rac1 activation, there was no change in total and partially activated levels of *β*1 integrin. However, significantly more fully activated *β*1 integrin was detected in the control but not Rac1-depleted PC-3 cells. For simplicity, the results shown are from one siRNA; however, near identical results were obtained with alternate siRNAs.

Figure 5(b) demonstrates that the decrease in CCL2-stimulated *β*1 integrin activity due to Rac1 depletion can be rescued by expression of an RNAi-insensitive Rac1 GTPase. Similarly, depletion of RhoG GTPase led to a significant decrease in CCL2-induced active *β*1 integrin expression as compared to scrambled control. Expression of a siRNA-resistant RhoG led to a significant increase in *β*1 integrin activation. In both cases, the RNAi-insensitive GTPases restored CCL2 activation of *β*1 integrin to levels comparable to the control cells. 

## 4. Discussion

The Rho GTPases comprise a subfamily of the Ras superfamily of monomeric GTP-binding proteins [21]. Like Ras, the Rho proteins transiently move from an inactive to active to an inactive state via the GTPase cycle. This cycle is controlled by a number of regulatory proteins, which in turn regulate Rho signal transduction via effector proteins [37–41]. This coordinate regulation of the Rho proteins allows for cytoskeletal reorganization leading to changes in cell shape and motility [12, 42]. Overexpression and/or aberrant activation of individual Rho GTPases has been shown in a number of cancers and is thought to drive metastatic progression [15]. Although one Rho protein may be the predominant GTPase in a cancer, other GTPases must also become active to reorganize the actin cytoskeleton and drive migration. 

RhoC GTPase is expressed in several cancers and promotes metastasis [43–52]. Previously, we demonstrated that RhoC GTPase expression and activation is required for PCa invasion [16, 18–20]. When RhoC expression or activation is downregulated, the PCa cells undergo Rac GTPase-mediated EMT [16, 18]. Decreased Rho expression or activity leads to increased expression and sustained activity of Rac1. Furthermore, Rac expression and activation was found to be required for tumor cell diapedesis across a human BMEC layer. We believe that together RhoC and Rac are needed to drive PCa extravasation from the vasculature into the bone marrow environment. 

There are four members of the Rac branch of the Rho subfamily: Rac1, Rac2, Rac3, and RhoG. Rac1 and Rac3, but not Rac2, are shown to have increased expression in PCa, while expression of RhoG has not been examined [33]. In the current study, we set out to determine the individual roles of Rac1, Rac3, and RhoG in tumor cell diapedesis. Rac1 levels are significantly higher than either Rac3 or RhoG suggesting that it is the predominant Rac GTPase in these cells. The relative levels of Rac1 and Rac3 in PCa are similar to what has been shown in glioblastoma cells [26].

Rac3 GTPase has clearly been shown to be involved in adhesion of tumor and normal cells of neural origin [24, 26]. Normal and malignant prostate has a neuroendocrine component; therefore, the question arises if Rac3 expression plays a role in neuroendocrine differentiation of PCa [53–55]. We have clear AFM data that implicates Rac3 in binding of PCa cells to fibronectin and to a lesser extent, collagen I (unpublished data). Binding to laminin would be the next logical choice to examine. This aspect may also begin to explain the apposing effect that Rac3 has on Rac1 and transendothelial cell migration. We found that downregulation of Rac3 led to an increase in total Rac activity, independent of an increase in total Rac protein levels. A similar observation was made previously; downregulation of RhoC increased Rac1 activity [16, 18]. However, this was accompanied by an increase in total Rac1 protein. 

Expression of RhoG is found ubiquitously throughout the body, but its expression in PCa has not been studied. We found that RhoG, although expressed in low levels, has an effect on total Rac activation. Inhibition of RhoG led to a significant decrease in Rac activation, but diapedesis was slightly increased. In contrast, CCL2-stimulation of PCa cells transfected with siRNA specific for RhoG significantly decreased diapedesis. Coexpression of a siRNA-resistant RhoG led to restoration of the cells ability to cross the endothelial cell layer. Furthermore, depletion of RhoG led to a significant decrease in CCL2-stimulated Rac activation, suggesting that CCL2 activates Rac1. Expression of a fast cycling RhoG in Rac1-depleted cells did not rescue the cells ability to undergo diapedesis. This also suggests that Rac1-mediated diapedesis may be regulated through direct activation of Rac1 or indirectly via RhoG GTPase and the different effects that RhoG has on PCa diapedesisis intriguing. Without CCL2 stimulation, RhoG appears to act like Rac3 and limit diapedesis, even after decreasing total Rac activation. Upon CCL2 stimulation, RhoG appears to play a role in activating Rac1 thereby decreasing diapedesis. This may suggest a specific RhoG GEF(s) that are activated by CCL2. Also of interest is the fact that ectopic expression of an RNAi-insensitive RhoG led to a significant decrease in unstimulated diapedesis suggesting a balance of RhoG expression required for migration. 

Our results measuring BMEC stiffness using AFM showed specific differences that were consistent over a large array of experimental attempts suggesting a specific biological interaction. The PC-3/BMEC interaction is of particular interest; PC-3 cells, whether adhered to substrate or attached to a BMEC, maintain a constant measured elastic modulus. The BMECs, however, when in contact with a PCa cell consistently, undergo a 30% decrease in stiffness. This apparent conferred decrease in stiffness points to a change in the internal cytoskeletal architecture of the BMEC. Depletion of Rac1 in the PC-3 cells led to a significant decrease in the strength of binding to the BMECs. The elasticity of the BMEC cell was not decreased when bound to a Rac1-depleted PC-3 cell indicating a Rac1-mediated interaction between the two cells. This interaction may be due, at least in part, to binding mediated by *β*1 integrins. We demonstrate that unstimulated PC-3 cells have partially activated *β*1 integrins that become fully activated upon CCL2 stimulation and activation of Rac1. Clearly, *β*1 integrins are required for binding of PCa cells to extracellular matrix [7, 19, 20, 56, 57]. Studies in the literature suggest a role for *β*1 integrins in binding PCa to BMECs [6, 7, 58, 59]. One report suggests that the use of a *β*1 integrin-blocking antibody did not affect PC-3 cell binding to the human bone marrow endothelial cell line HBME-1 but was responsible for mediating PCa interactions with fibronectin [7]. However, other studies show a role for *β*1 integrins in binding to other bone marrow endothelial cells [58, 59]. Rho GTPases such as Rac1 are implicated in bidirectional signaling with integrins activating Rho proteins and the active Rho proteins promoting integrin dimer activation increasing binding strength [36, 60, 61]. 

These mechanisms are similar to leukocyte diapedesis, where, after initial binding, the interaction between the leukocyte and endothelial cell increases leading to dynamic cytoskeletal changes and endothelial cell retraction [62, 63]. Although this has been suggested for PCa diapedesis, this is the first time this has been shown experimentally. A complete understanding of how these different Rac proteins are activated and how they contribute to tumor cell diapedesis may have profound implications for any strategies targeting the extravasation process.

##  Conflict of Interests

The authors have no conflict of interests to disclose.

## Supplementary Material

Supplemental Figure 1 is semi-quantitative PCR analysis for expression of Rac1, Rac3 and RhoG GTPases in the PC-3 prostate cancer cell line. Primers for each PCR are listed in Supplemental Table 1. Represented is a virtual gel produced by semi-quantitative PCR on an Evocycler using FastStart SYBR Green. Each sample is normalized to GAPDH expression from the corresponding sample giving relative band intensities corresponding to expression levels.
Shown in supplemental Figure 2 is the effect of Rac GTPases in the C4-2 LNCaP series prostate cancer cell line. Panel a. Rac isoform expression in C4-2 cells. C4-2 mRNA was harvested and SYBR green-based qPCR performed using primers specified in Supplemental Table 1. Relative expression levels were normalized to GAPDH expression from the corresponding sample and expressed as arbitrary units (a.u.). Panel b. are results of a diapedesis assay after depleting Rac isoforms in C4-2 cells. BMECs were layered onto a Matrigel coated filter and allowed to form a monolayer, 0.5 ml of a suspension of 3.75 x 105 C4-2 cells/ml were added to the BMECs and allowed to undergo diapedesis for 24 h. Compared with untransfected or scrambled controls, depletion of Rac1 or RhoG led to a significant decrease in diapedesis while depletion of Rac3 led to a significant increase in diapedesis. Panel c. C4-2 cells were treated with 100 ng/ml CCL2 in a diapedesis assay as described. Control untransfected (UT) and siRNA control (siScr) cells were compared with untreated/untransfected (UN/UT) C4-2 cells. Cells ability to under go CCL-2 stimulated diapedesis after depletion of Rac1 and RhoG or inhibition of total Rac with iRac was compared to UT and siScr. Rescue experiments were performed by introduction of a siRNA-resistant RhoG led to a significant reversion of RhoG inhibition of diapedesis. For both panels b and c (∗) signifies a significant difference between siRNA transfected cells and stimulated controls while (^) signifies a significant difference between siRNA transfected and rescued cells.
Finally, supplemental Figure 3 is a comparison of elasticity in PC-3 and BMECs. PC-3 cells transfected with either a control (siScr) or Rac1-specific SMARTpool siRNA(2) were grown in a monolayer. BMECs were layered onto the PC-3 monolayer, allowed to attach for 30 min and then the elasticity determined by AFM. BMECs in contact with control PC-3 cells had a significant increase in elasticity (∗P<.001). Represented are means ± S.D.

## Abbreviations

PCa: Prostate cancer
Rho: Ras homology
dnRho: Dominant negative Rho
shRNA/siRNA: Small hairpin RNA/small inhibitor RNA
FBS: Fetal bovine serum
BMEC: Bone marrow endothelial cell
ECM: Extracellular matrix
AFM: Atomic force microscopy.
