# Supplementary figures and images for: Individual Rac GTPases Mediate Aspects of Prostate Cancer Cell and Bone Marrow Endothelial Cell Interactions
Source: J Signal Transduct. 2011 Jun 27;2011:541851. doi: 10.1155/2011/541851 (PMC3135208; doi:10.1155/2011/541851)

**Rac1**

**Rac3**

**RhoG**

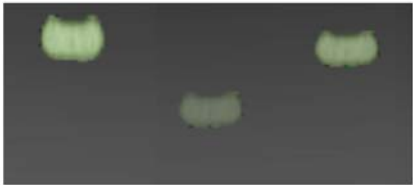

Supplement: Supplementary file 1 — Supplemental Figure 1 is semi-quantitative PCR analysis for expression of Rac1, Rac3 and RhoG GTPases in the PC-3 prostate cancer cell line. Primers for each PCR are listed in Supplemental Table 1. Represented is a virtual gel produced by semi-quantitative PCR on an Evocycler using FastStart SYBR Green. Each sample is normalized to GAPDH expression from the corresponding sample giving relative band intensities corresponding to expression levels. Shown in supplemental Figure 2 is the effect of Rac GTPases in the C4-2 LNCaP series prostate cancer cell line. Panel a. Rac isoform expression in C4-2 cells. C4-2 mRNA was harvested and SYBR green-based qPCR performed using primers specified in Supplemental Table 1. Relative expression levels were normalized to GAPDH expression from the corresponding sample and expressed as arbitrary units (a.u.). Panel b. are results of a diapedesis assay after depleting Rac isoforms in C4-2 cells. BMECs were layered onto a Matrigel coated filter and allowed to form a monolayer, 0.5 ml of a suspension of 3.75 x 10^5^ C4-2 cells/ml were added to the BMECs and allowed to undergo diapedesis for 24 h. Compared with untransfected or scrambled controls, depletion of Rac1 or RhoG led to a significant decrease in diapedesis while depletion of Rac3 led to a significant increase in diapedesis. Panel c. C4-2 cells were treated with 100 ng/ml CCL2 in a diapedesis assay as described. Control untransfected (UT) and siRNA control (siScr) cells were compared with untreated/untransfected (UN/UT) C4-2 cells. Cells ability to under go CCL-2 stimulated diapedesis after depletion of Rac1 and RhoG or inhibition of total Rac with iRac was compared to UT and siScr. Rescue experiments were performed by introduction of a siRNA-resistant RhoG led to a significant reversion of RhoG inhibition of diapedesis. For both panels b and c (∗) signifies a significant difference between siRNA transfected cells and stimulated controls while (^) signifies a significant difference [file 541851.f1.pdf]

**a.**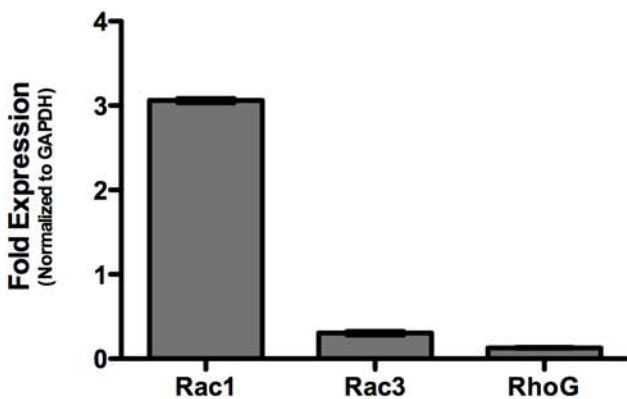**b.**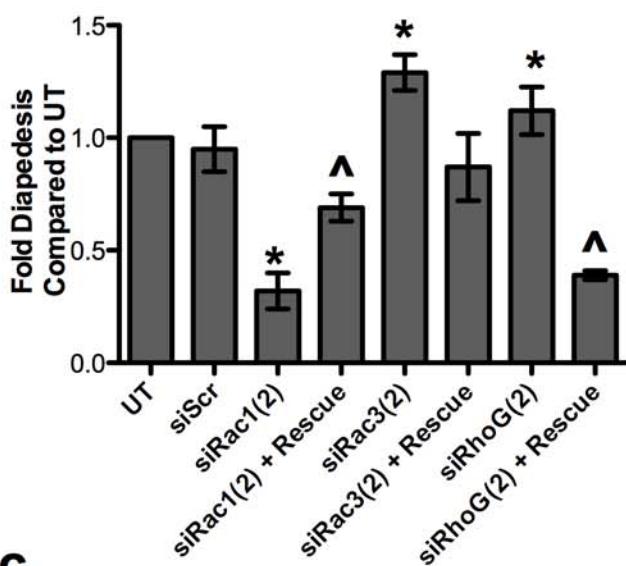**c.**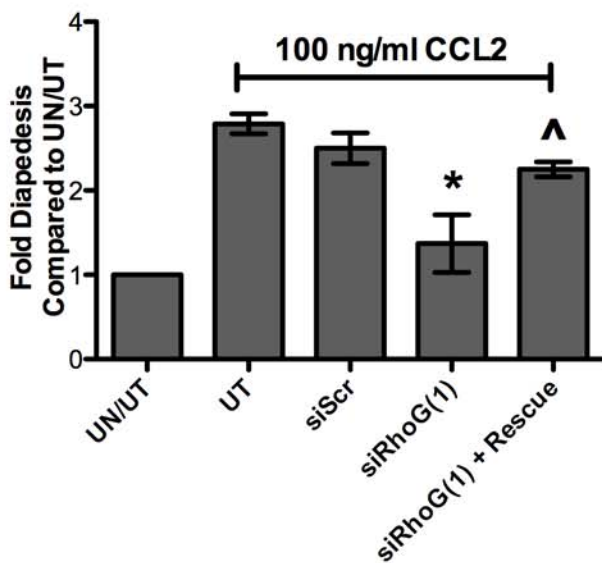

Supplement: Supplementary file 2 [file 541851.f2.pdf]

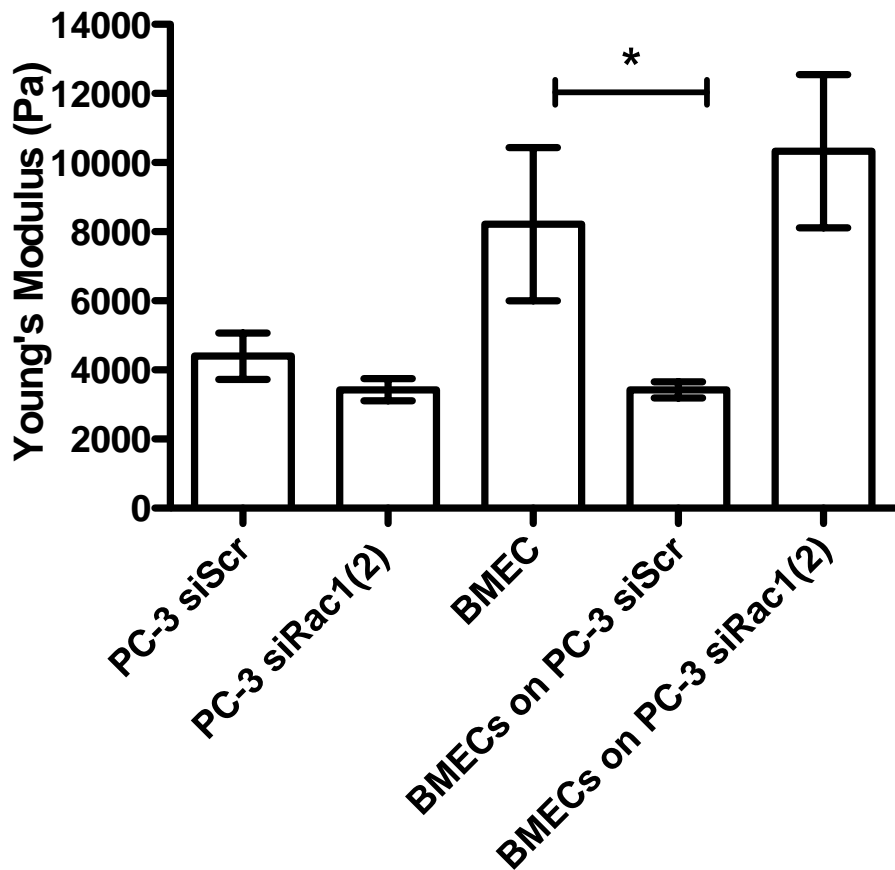

Supplement: Supplementary file 3 [file 541851.f3.pdf]
