# Supplementary material for: Individual Rac GTPases Mediate Aspects of Prostate Cancer Cell and Bone Marrow Endothelial Cell Interactions
Source: J Signal Transduct. 2011 Jun 27;2011:541851. doi: 10.1155/2011/541851 (PMC3135208; doi:10.1155/2011/541851)
Supplement: Supplementary file 4 [file 541851.f4.pdf]

## Supplemental Table 1.

### Primers for qPCR:

| Primer id | Primer sequence |
|---|---|
| Rac1 Fwd | 5’-AAG AGA AAA TGC CTG CTG TTG TAA-3’ |
| Rac1 Rev | 5’-GCG TAC AAA GGT TCC AAG GG-3’ |
| Rac3 Fwd | 5’-GGG AAG ACA TGC TTG CTG ATC-3’ |
| Rac3 Rev | 5-CCT CCT GAC CCG CTG TGT-3’ |
| RhoG Fwd | 5’-GTT TCT CCA TTG CCA GTC CG-3’ |
| RhoG Rev | 5’-CAC CGT CGG TCT CCA CAC G-3’ |
| GAPDH Fwd | 5’-GTG AAG GTC GGA GTC AAC G-3’ |
| GAPDH Rev | 5’-GGT GAA GAC GCC AGT GGA CTC-3’ |

### Primers for Evocycler:

| Primer id | Primer sequence |
|---|---|
| Rac1 Fwd | 5’-GCG CCC CGC CGC CCG CAA GC-3’ |
| Rac1 Rev | 5’-GTC GCG GGA CGG CGG CGG CG-3’ |
| Rac3 Fwd | 5’-TGC GGC GCC GGG CAT TTC TC-3’ |
| Rac3 Rev | 5’-GTT CAC GCA CCA CCA GCC GC-3’ |
| RhoG Fwd | 5’-AGG GCC AGG CGC CCA TCA CA-3’ |
| RhoG Rev | 5’-ACA AGC GGC TCC GAC AGG CC-3’ |
| GAPDH Fwd | 5’-TGG CCC CTC CGG GAA ACT GT-3’ |
| GAPDH Rev | 5’-GTA GGG ACG GAG ATG ACC GC-3’ |
